# Orbitofrontal Cortex Encodes Preference for Alcohol

**DOI:** 10.1523/ENEURO.0402-19.2020

**Published:** 2020-07-15

**Authors:** John S. Hernandez, David E. Moorman

**Affiliations:** 1 Neuroscience and Behavior Graduate Program, University of Massachusetts Amherst, Amherst, MA 01003; 2 Department of Psychological and Brain Sciences, University of Massachusetts Amherst, Amherst, MA 01003

**Keywords:** alcohol use disorder, dependence, electrophysiology, instrumental, orbital cortex, prefrontal cortex

## Abstract

Orbitofrontal cortex (OFC) plays a key role in representation and regulation of reward value, preference, and seeking. OFC function is disrupted in drug use and dependence, but its specific role in alcohol use disorders has not been thoroughly studied. In alcohol-dependent humans OFC activity is increased by alcohol cue presentation. Ethanol (EtOH) also alters OFC neuron excitability *in vitro*, and OFC manipulation influences EtOH seeking and drinking in rodents. To understand the relationship between OFC function and individual alcohol use, we recorded OFC neuron activity in rats during EtOH self-administration, characterizing the neural correlates of individual preference for alcohol. After one month of intermittent access to 20% EtOH, male Long–Evans rats were trained to self-administer 20% EtOH, 10% EtOH, and 15% sucrose. OFC neuronal activity was recorded and associated with task performance and EtOH preference. Rats segregated into high and low EtOH drinkers based on homecage consumption and operant seeking of 20% EtOH. Motivation for 10% EtOH and sucrose was equally high in both groups. OFC neuronal activity was robustly increased or decreased during sucrose and EtOH seeking and consumption, and strength of changes in OFC activity was directly associated with individual preference for 20% EtOH. EtOH-associated OFC activity was more similar to sucrose-associated activity in high versus low EtOH drinkers. The results show that OFC neurons are activated during alcohol seeking based on individual preference, supporting this brain region as a potential substrate for alcohol motivation that may be dysregulated in alcohol misuse.

## Abstract (precis)

Orbitofrontal cortex (OFC) plays a key role in representation and regulation of reward value, preference, and seeking. OFC function is disrupted in drug use and dependence, but its specific role in alcohol use disorders has not been thoroughly studied. In alcohol-dependent humans OFC activity is increased by alcohol cue presentation. Ethanol (EtOH) also alters OFC neuron excitability *in vitro*, and OFC manipulation influences EtOH seeking and drinking in rodents.

## Significance Statement

Understanding how alcohol preference manifests in the brain is important for understanding use and misuse. We trained rats to self-administer alcohol and sucrose, as a positive preference control. During self-administration, we recorded the activity of neurons in the orbitofrontal cortex (OFC), an area previously associated with reward motivation and drug use. OFC neuronal activity was aligned with behavioral alcohol preference, both at a population level and on an individual basis. OFC activity of high alcohol-preferring rats during alcohol seeking and consumption was similar to that seen during sucrose-associated behaviors. These data provide evidence that the OFC is a key region underlying individual alcohol preference and suggests further scrutiny of OFC in the context of alcohol use disorder.

## Introduction

The orbitofrontal cortex (OFC) regulates reward seeking and cognitive strategies associated with optimizing outcomes (Dalley et al., 2004; Kringelbach and Rolls, 2004; O'Doherty, 2007; Mainen and Kepecs, 2009; Schoenbaum et al., 2009; Balleine et al., 2011; Padoa-Schioppa, 2011; Wallis, 2011; Walton et al., 2011; McDannald et al., 2014a; Rudebeck and Murray, 2014; Izquierdo, 2017). OFC is activated during craving and seeking of drugs of abuse and in response to drug-associated cues (Garavan et al., 2000; Risinger et al., 2005; Baeg et al., 2009; Guillem et al., 2010, 2018; Guillem and Ahmed, 2018a). OFC hypoactivity is associated with impulsivity and drug use disorders (Whelan et al., 2012). Based on these and other results, OFC disruption has been hypothesized to be a major factor underlying drug addiction (London et al., 2000; Porrino and Lyons, 2000; Volkow and Fowler, 2000; Dom et al., 2005; Everitt et al., 2007; Winstanley, 2007; Schoenbaum and Shaham, 2008; Lucantonio et al., 2014; Fettes et al., 2017). However, only a subset of addiction-related studies has investigated the role of OFC in alcohol use.

There is some evidence for a role for OFC in alcohol motivation and dependence (Moorman, 2018). OFC activation in humans has been associated with alcohol-related craving (Myrick et al., 2004, 2008; Lukas et al., 2013; Schacht et al., 2013a,b, 2014). Connectivity between OFC and striatum is altered in abstinent alcoholics (Volkow et al., 2007). Chronic alcohol (ethanol, EtOH) results in cognitive deficits associated with OFC dysfunction (Lejuez et al., 2010; Badanich et al., 2011). EtOH consumption and reinstatement increases Fos and ΔFosB expression in rodent OFC (Li et al., 2010; Jupp et al., 2011). Acute EtOH *in vitro* inhibits OFC neuron excitability and synaptic function (Badanich et al., 2013b). In mice withdrawn from chronic EtOH vapor, OFC neurons display increases in spine density and basal excitability and a diminished inhibitory response to acute EtOH (McGuier et al., 2015; Nimitvilai et al., 2016). OFC lesions or DREADD inhibition increased alcohol drinking in rats and EtOH vapor-treated mice (den Hartog et al., 2016; Ray et al., 2018), and inactivation decreased context-induced reinstatement in rats (Bianchi et al., 2018).

Missing from these studies is an analysis of neural dynamics associated with alcohol seeking, and how these dynamics may vary across individuals. Alcohol motivation varies among human and animal subjects, and individuals exhibiting greater euphoric or stimulating effects of alcohol may be at greater risk for misuse and dependence (King et al., 2011; Sharko et al., 2013; Momeni and Roman, 2014; Spoelder et al., 2015; Moorman et al., 2016, 2017; Juarez et al., 2017). Given the strong association between OFC neuronal function and individual preferences for natural rewards, cocaine, and heroin (Tremblay and Schultz, 1999; Padoa-Schioppa and Assad, 2006; Schultz, 2010; Guillem and Ahmed, 2018b; Guillem et al., 2018), we predicted that differential OFC activity may reflect individual alcohol preferences. We characterized individual preference for EtOH in outbred Long–Evans rats and identified OFC correlates of this preference by recording neuronal activity during operant EtOH or sucrose seeking. Our results indicate that OFC neurons are strongly, but differentially, activated during EtOH and sucrose seeking, and that degree of activation is associated with individual EtOH preference.

## Materials and Methods

Male Long–Evans rats (*n* = 24; ∼200–300 g on arrival; Charles River Laboratories) were kept in temperature-controlled and humidity-controlled conditions under reversed light/dark cycle (7 A.M. off to 7 P.M. on). Water was available *ad libitum*, and rats were restricted to 25 g of rat chow given after daily operant sessions once they reached 300 g. All animal procedures were approved by the Institutional Animal Care and Use Committee at the University of Massachusetts Amherst and were conducted in compliance with the National Institutes of Health *Guide for the Care and Use of Animals*.

### Experimental design

**Figure 1.**
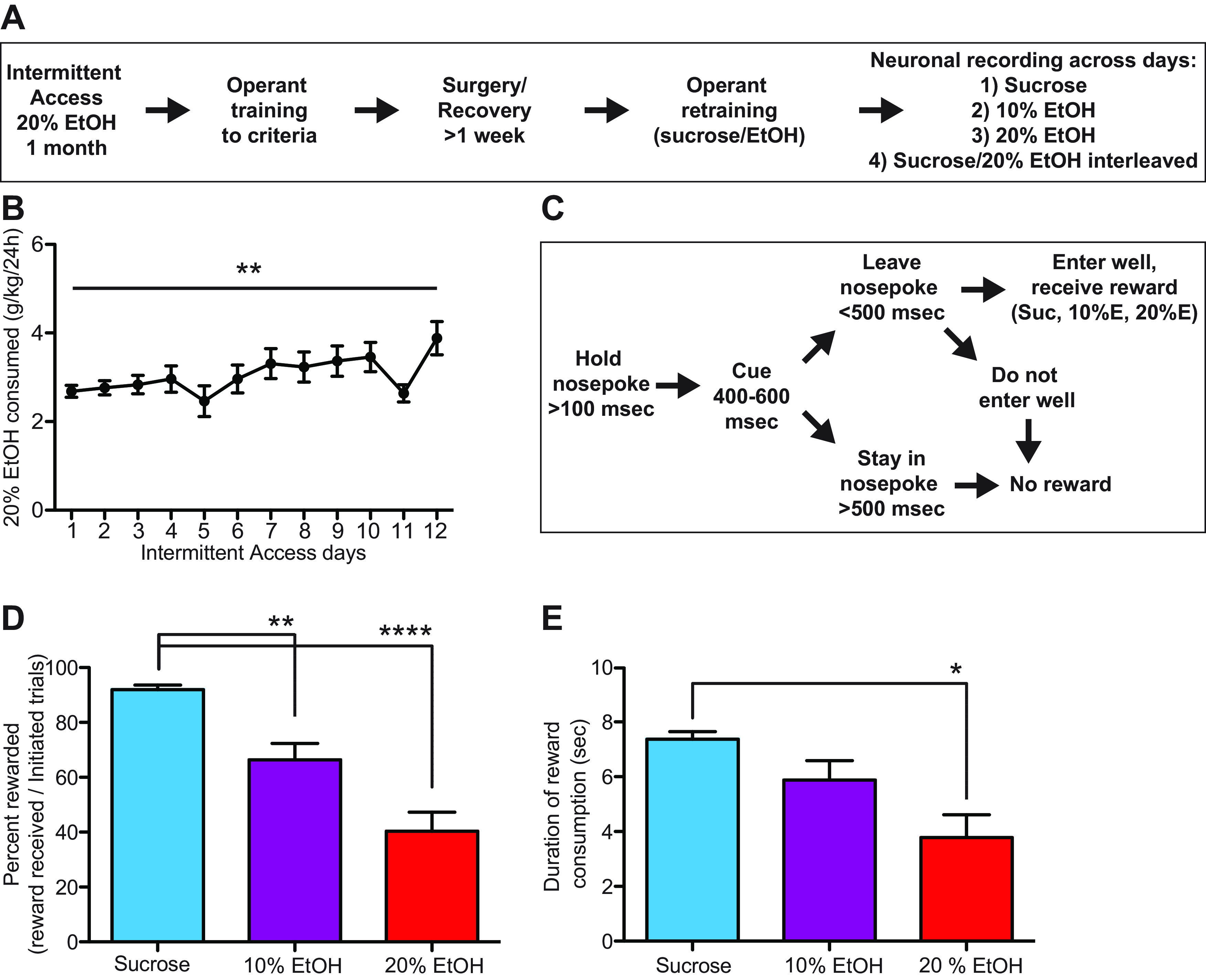
***A***, Experimental timeline. ***B***, Rats received 12-d homecage intermittent access to EtOH. Consumption escalated significantly over days. ***C***, Operant task diagram. Rats nosepoked to receive cues predicting sucrose or EtOH. Outcomes were consumed by licking a spigot in a reward port below the nosepoke. ***D***, Rats acquired significantly more sucrose (blue) than 10% EtOH (purple) or 20% EtOH (red) rewards. ***E***, Rats consumed significantly more sucrose than 20% EtOH based on duration of reward consumption; *p *<* 0.05, **p *<* 0.01, ****p *<* 0.0001.

Experimental design is shown in Figure 1*A*. Animals were trained to drink 20% EtOH (Fisher Scientific) for one month in their home cages using the intermittent access to EtOH paradigm (Wise, 1975; Simms et al., 2008; Moorman and Aston-Jones, 2009; Carnicella et al., 2014; Moorman et al., 2016, 2017), with *ad libitum* access to food and water. Rats were then trained to perform operant EtOH and sucrose seeking on an fixed ratio 1 (FR1) schedule (Fig. 1*C*). Behavioral testing was conducted in operant chambers (Med Associates) equipped with a house-light, nosepoke, and reward delivery port containing a spigot to deliver three rewards (15% sucrose, 10% EtOH, 20% EtOH) separately. Nosepokes, reward port entries, and licks were detected with infrared beam breaks, thereby reducing electrical noise for electrophysiological recording. Fluids were consumed at the spigot only (there was no collection well). Non-consumed sucrose or alcohol drained away from the reward delivery port to prevent fluid mixing. Rats were initially trained on FR1 nosepoke responses for a sucrose cue (5-kHz tone, duration 400–600 ms) and 0.1-ml sucrose delivery through the spigot. After rats reached a criterion of 85% successful trials (retrieving sucrose within 500-ms postcue onset), rats were prepared for surgical implantation of recording arrays. Homecage EtOH access was used to train rats to drink EtOH as well as to characterize individual EtOH preference in the absence of any additional demands that might be present in an operant context.

After recovery, rats were retrained on the nosepoke-cue-sucrose task for 2 d. We then recorded OFC neuron activity while rats performed nosepoke-cue-outcome tasks, where cue-outcome pairings were 5-kHz tone–15% sucrose, 1-kHz tone–20% EtOH, or 10-kHz tone–10% EtOH. Recording sessions consisted of either blocked trials (where all trials per session/day were one cue-outcome pairing) or interleaved trials (where sucrose and 20% EtOH trials were pseudorandomly interleaved). Because it is difficult to reliably claim stable recording of the same neuron across multiple days using microwire arrays, we included the interleaved sessions so that we could compare the activity of OFC neurons during both EtOH and sucrose conditions. All trials were self-initiated, and animals were free to consume or not consume rewards after trial initiation. All sessions lasted 1 h. During blocked sessions, only one cue-outcome set of trials was presented each day, mitigating concerns that previous blocks may have influenced expectations on the current recording session. For each cue-outcome pairing, OFC activity was recorded two times (once per day for 2 d). Activity from the session with the best quality recording (signal-to-noise, numbers of neurons) was included in analysis. All rats received at least one (typically three or more) days of 10% or 20% EtOH self-administration before OFC recording, mitigating concerns that OFC activity on EtOH self-administration days reflected a deviation from expected sucrose reward.

### Surgery

Surgical methods were similar to our previous work (Moorman and Aston-Jones, 2014, 2015). Rats were given 100 mg/l minocycline HCl (Henry Schein Medical) *ad libitum* 2 d before and 5 d after array implantation. Meloxicam (Metacam; Henry Schein Medical) was administered 1.36 mg/kg (subcutaneously). Under isoflurane anesthesia (1.5−2.5%), custom-made, static arrays of 32 recording electrodes (50-μm nichrome wires, 200-μm spacing center-to-center) were implanted unilaterally in OFC. Arrays spanned from medial OFC (mOFC: A/P 3.6–4.6 mm, M/L 0.7 mm, D/V −5.0 to −5.2 mm from bregma) to lateral OFC (lOFC: A/P 3.6–4.6 mm, M/L 2.6–3.0 mm, D/V −5.0 to −5.2 mm from bregma).

### Electrophysiological recordings

Electrophysiological recordings were performed using a Digital Lynx system (Neuralynx). OFC neurons were recorded on each blocked and interleaved session for 2 d. Wideband signals were filtered 300–3000 Hz and thresholded to identify well isolated action potentials (≥4 SD from mean of peak height of noise band), which were manually sorted in Offline Sorter (Plexon). Well-isolated units that fired throughout each recording session were included in analyses.

### Histology

After the final recording, rats were anesthetized with 1.5–2.5% isoflurane and constant current (25 μA) was delivered to each recording wire for 15 s to produce lesions to mark the tips of recording electrodes. One day later, rats were perfused with 0.9% NaCl solution followed by 4% paraformaldehyde. Brains were postfixed overnight with 4% paraformaldehyde and cryoprotected in a 20% sucrose/0.1% sodium azide solution. Forty-micrometer sections were stained with neutral red to confirm electrode placement.

### Data analysis

Rats were identified as high drinkers (HD) or low drinkers (LD) if mean EtOH consumption on the final 3 d of homecage intermittent access was greater or less than 3.5 g/kg/24 h, respectively (George et al., 2012; Momeni and Roman, 2014; Spoelder et al., 2015). Operant behaviors analyzed included total number of rewards received, percent rewarded trials, average duration of reward consumption, and latency to acquire reward. Behavioral and neurophysiological data were analyzed using standard parametric or nonparametric tests depending on normality, using Prism (GraphPad) or custom analyses in MATLAB (MathWorks).

Neuronal activity was grouped in 50-ms bins and aligned to task events. Spike density functions (SDFs) were generated by Gaussian smoothing. Population activity plots were made by *z* score normalizing event-related neuronal activity against baseline activity preceding trial initiation. OFC response strength was calculated with the index:

$$\frac{\left(\text{Behavioral epoch activity }–\text{ baseline activity}\right)}{\left(\text{Behavioral epoch activity }+\text{ baseline activity}\right)},$$

where activity was number of spikes during a given behavioral epoch or a baseline epoch of equal duration sampled, on a trial-by-trial basis, from the pre-trial-initiation intertrial interval. Three main test epochs were studied along with trial-matched baseline epochs: postcue/preseeking (cue onset to cue + 100 ms), reward seeking (400-ms prereward receipt to reward receipt, measured as first rewarded lick), and reward consumption (first rewarded lick to last rewarded lick). Wilcoxon signed-rank tests were used to measure significant shifts from zero in distribution plots for all indices (Roesch et al., 2012; Takahashi et al., 2013). Each epoch was aligned to an event that, because of the self-paced nature of the task, was variable from trial to trial. Although rats performed the task consistently across trials, the inherent variability in the trial initiation, nosepoke-exit, and reward consumption, permitted analyses focused specifically on those events. All analyses were considered significant at α = 0.05.

## Results

### Rats engaged in EtOH and sucrose seeking and consumption, preferring sucrose more than EtOH, and 10% EtOH more than 20% EtOH

Across all rats 20% EtOH consumption escalated slightly but significantly during homecage intermittent access (*H*_(11)_ = 25.58, *p* = 0.008, Kruskal–Wallis; Fig. 1*B*). During operant testing, rats nosepoked to receive a tone cue predicting one of three outcomes (20% EtOH, 10% EtOH, or 15% sucrose; Fig. 1*C*). Following cue presentation, rats withdrew from the nosepoke and received reward from a spigot directly below the nosepoke. Of the 24 rats tested in homecage intermittent access, 16 rats were used for neurophysiological recording during operant sucrose and 20% EtOH seeking sessions, and 12/16 rats were also used for recording during 10% EtOH sessions.

Reward motivation, measured as percent rewarded trials (number of rewarded trials divided by number of initiated trials), was significantly influenced by reward type (*H*_(2)_ = 26.54, *p* *<* 0.0001; Fig. 1*D*). Rats received significantly more sucrose than 20% EtOH rewards [*p* *<* 0.0001, Dunn’s multiple comparisons test (MCT)] and 10% EtOH rewards (*p* = 0.007). Rats also received fewer 20% EtOH than 10% EtOH rewards, although this difference was not significant. We also quantified consumption via licking duration, measured from first lick during reward delivery to the final lick of that trial, for each outcome as a measure of preference. As with reward acquisition, reward consumption (Fig. 1*E*) was significantly different across conditions (*H*_(2)_ = 9.16, *p* *=* 0.010) and was greater for sucrose than 20% and 10% EtOH, with the difference between sucrose and 20% EtOH being significant (*p* = 0.008, Dunn’s MCT).

We also measured response latencies to verify that our analysis epochs captured non-overlapping neural correlates of behavior. Median latencies from cue onset to nosepoke exit were 110 ms (sucrose), 210 ms (10% EtOH), and 170 ms (20% EtOH), so our “cue” analysis epoch (cue onset time to cue + 100 ms) was focused on the cue-evoked decision and initiation of the nosepoke exit response. Median latencies from nosepoke exit to first reward lick were 890 ms (sucrose), 960 ms (10% EtOH), and 1355 ms (20% EtOH). Thus, the “seeking” analysis epoch (400 ms before first rewarded lick to first rewarded lick) was focused exclusively on reward-seeking behaviors and did not overlap with nosepoke exit or cue presentation.

### OFC neuronal activity was strongly but differentially altered during sucrose and EtOH seeking and consumption

**Figure 2.**
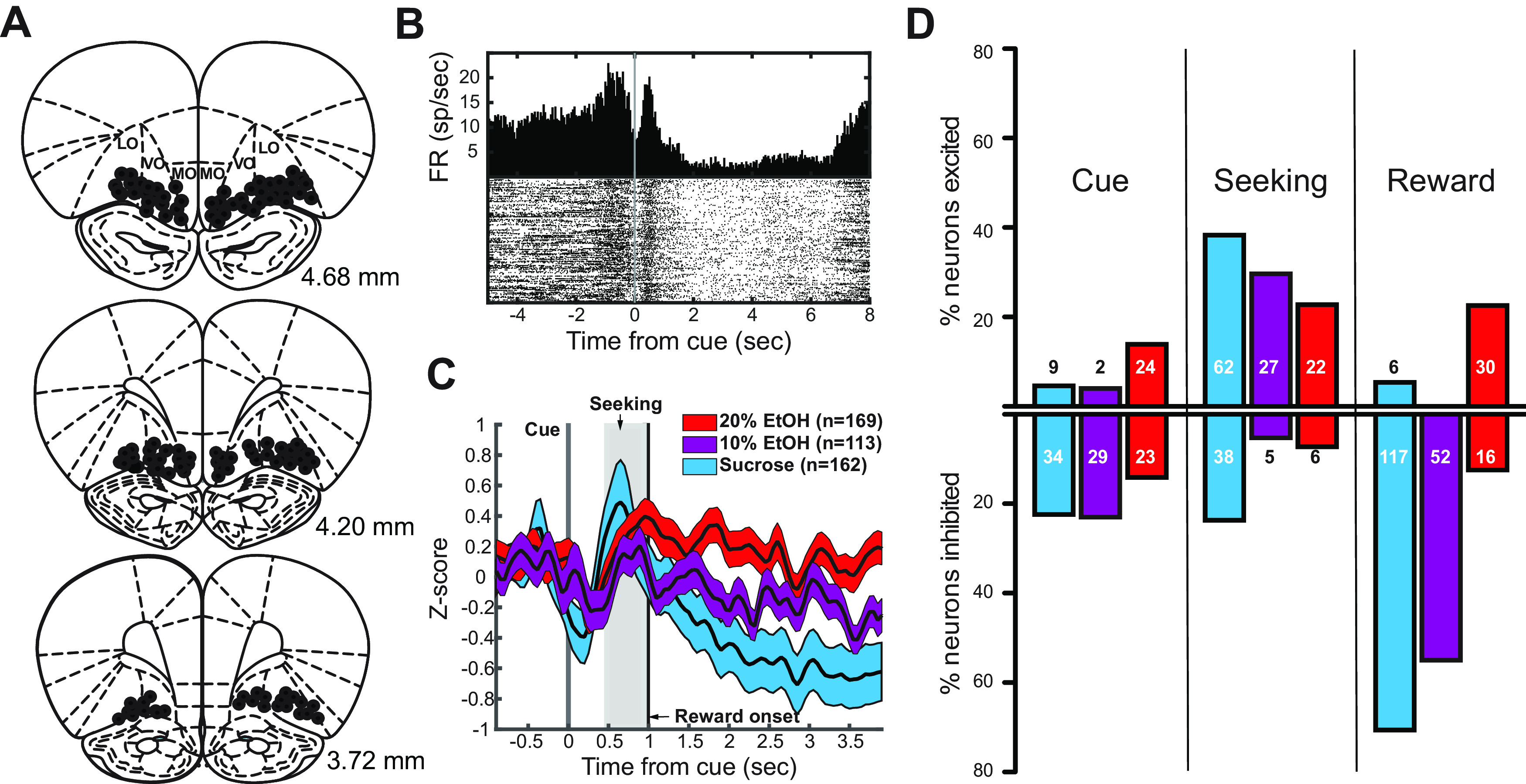
***A***, Recording sites in the OFC (black circles) based on lesions made postrecording. ***B***, Example activity from OFC neuron recorded during a sucrose session. Rasters (bottom) and 50-ms bin histogram (top) show the prominent inhibition during cue presentation followed by excitation during reward seeking and inhibition during reward consumption. ***C***, Z-scored average OFC activity with standard error confidence intervals (CIs) across all neurons aligned on cue presentation in sucrose (blue CI), 10% EtOH (purple CI), and 20% EtOH (red CI) sessions. ***D***, Numbers of neurons significantly excited or inhibited during each epoch in sucrose (blue), 10% EtOH (purple), and 20% EtOH (red) sessions.

**Figure 3.**
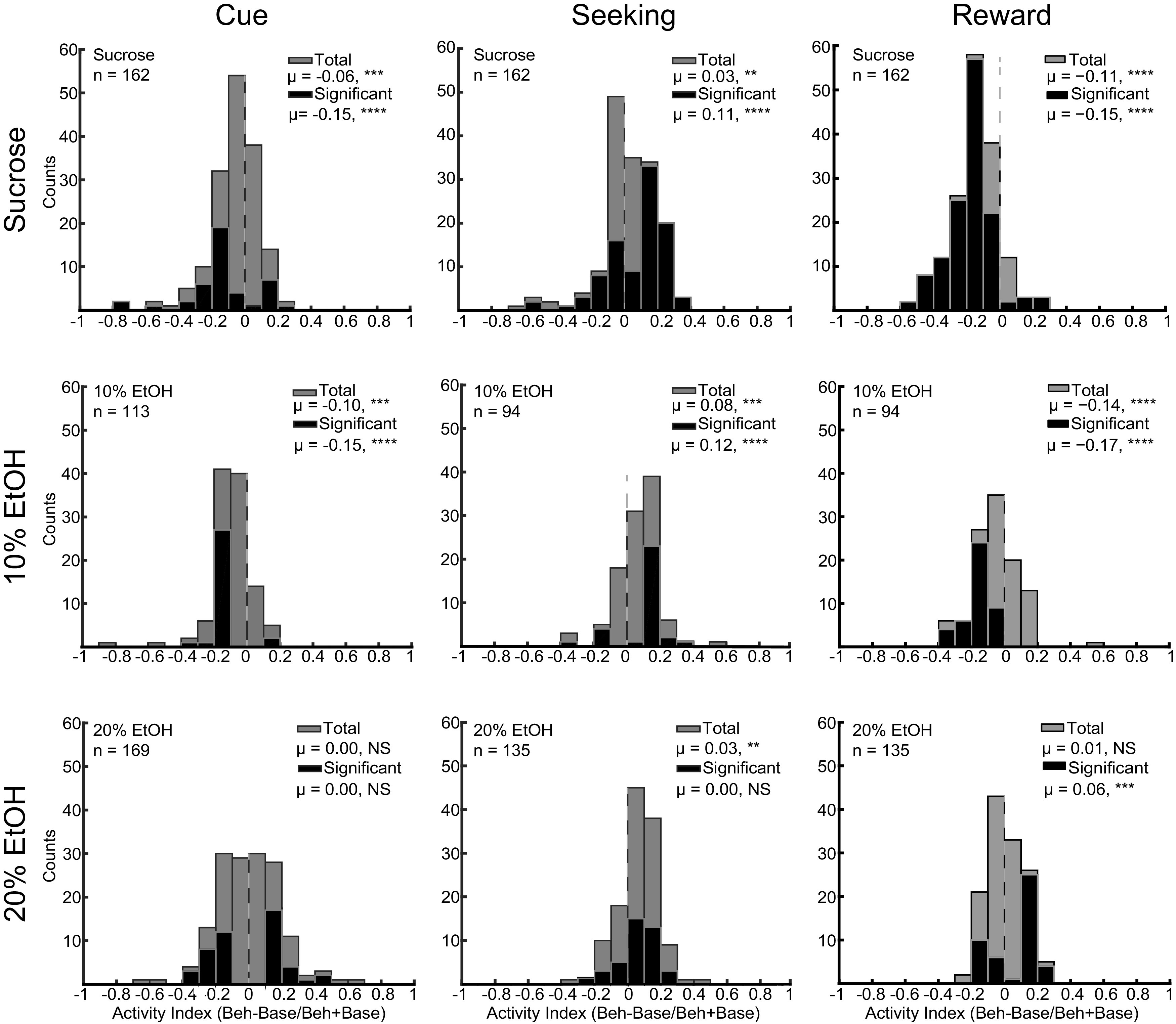
Index calculations for individual OFC neuron activity during cue presentation (left column), reward seeking (middle column), and reward consumption (right column) during sucrose (top row), 10% EtOH (middle row), and 20% EtOH (bottom row) sessions. Indices calculated as in Materials and Methods. Beh = behavioral epoch; Base = baseline epoch. NS not significant; **p *<* 0.01, ***p *<* 0.001, ****p *<* 0.0001.

Based on histologic characterization of postrecording lesion sites, neurons were recorded from OFC but spanned the extent of mOFC, ventral OFC (vOFC), and lOFC (Fig. 2*A*). OFC neuronal activity was analyzed during three epochs: cue presentation, reward seeking, and reward consumption (Materials and Methods). During sucrose sessions, OFC neurons were suppressed during cue presentation, activated during reward seeking, and again suppressed during reward consumption (Fig. 2*B–D*, blue), consistent with our previous report (Moorman and Aston-Jones, 2014), although there were examples of individual neurons exhibiting different profiles (Figs. 2*D*, 3). During 10% EtOH trials, OFC neurons exhibited a similar pattern to that seen during sucrose seeking and consumption (Figs. 2*C*,*D*, purple, 3), but with slightly reduced proportions of responses and a stronger bias toward excitation during seeking. During 20% EtOH sessions, OFC neuronal activity was significantly increased and decreased, but the proportions of excitation versus inhibition were strikingly different during cue presentation and reward consumption (Figs. 2*C*,*D*, red, 3). This was particularly salient during consumption in which the strong bias toward inhibition seen in sucrose and 10% EtOH was reversed and more neurons exhibited excitation. There were no significant differences in baseline activity, measured during the intertrial interval, across sessions (see below).

### OFC neuronal response properties were consistent with the population, but overall heterogeneous

The differences in proportions of significantly upregulated/downregulated neurons shown in Figure 2 were consistent with distributions of response indices of single neurons shown in Figure 3. During the cue presentation epoch (Fig. 3, left column), neuronal activity was significantly suppressed in both sucrose (significant neurons (sig): z = −4.25, *p* < 0.0001; all neurons (all): z = −5.00, *p* = 0.0008; Wilcoxon) and 10% EtOH (sig: z = −7.12, *p* = 0.0004; all: z = −3.94, *p* < 0.0001) trials, whereas activity was not significantly biased in 20% EtOH trials (sig: z = −0.46, *p* = 0.8; all: z = −0.29, *p* = 0.7). During reward seeking (Fig. 3, center column), activity was biased toward excitation in sucrose (sig: z = 4.77, *p* < 0.0001; all: z = 3.16, *p* = 0.008), 10% EtOH (sig: z = 3.59, *p* = 0.0004; all: z = 5.46, *p* < 0.0001) and 20% EtOH (sig: z = 1.55, *p* = 0.1; all: z = 4.29, *p* = 0.006) trials. During reward consumption (Fig. 3, right column), activity was significantly reduced during sucrose (sig: z = −8.94, *p* *<* 0.0001; all: z = −7.56, *p* < 0.0001; Wilcoxon) and 10% EtOH consumption (sig: z = −7.32, *p* *<* 0.0001; all: z = −6.27. *p* < 0.0001). In contrast, during 20% EtOH consumption, few neurons were significantly suppressed, and more neurons actually exhibited increased activation. This excitation bias was significant for neurons with significant changes in firing rates (z = 3.06, *p* = 0.0002) but not across the whole population (z = 0.87, *p* *=* 0.4). Differences between distributions were significant (cue: *F*_(2,433)_ = 7.7629, *p* = 0.022; seeking: *F*_(2,381)_ = 12.24, *p* = 0.027; consumption: *F*_(2,388)_ = 49.229, *p* < 0.0001; Kruskal–Wallis). These differences were driven by significant differences in distribution of responses to sucrose versus 20% EtOH (cue: *p* = 0.0187, seeking: *p* = 0.023, consumption: *p* < 0.0001) and, during reward consumption, 10% EtOH versus 20% EtOH (cue: *p* = 0.3337, seeking: *p* = 0.17, consumption: *p* < 0.0001), but not sucrose versus 10% EtOH (cue: *p* = 1, seeking: *p* = 1, consumption: *p* = 0.8931). When isolating significantly modulated neurons, distributions were significantly different (cue: *F*_(2,121)_ = 8.759, *p* = 0.0125; seeking: *F*_(2,179)_ = 11.06, *p* = 0.004; consumption: *F*_(2,221)_ = 63.58, *p* < 0.0001; Kruskal–Wallis). These significant differences were driven by differences in distribution of responses to sucrose and 20% EtOH (cue: *p* = 0.032, seeking: *p* = 0.0366, consumption: *p* < 0.0001) as well as 10% EtOH and 20% EtOH (cue: *p* = 0.042, seeking: *p* = 0.049, consumption: *p* < 0.0001). Thus, both in populations of neurons with significantly different firing rates, and across the population as a whole, OFC neurons exhibited differential outcome classification, based on proportions of excitatory and inhibitory responses, at different task stages.

Although Figures 2, 3 provide a summary of the overall output of OFC combined across neurons, Figure 3 also shows significant heterogeneity across neuronal responses. To further characterize the degree to which OFC neurons conformed to specific patterns as shown, for example in Figure 2*B*,*C*, we characterized, for each neuron, whether the neuron exhibited significant excitation (+1), inhibition (−1), or neither (0) during each of the three epochs (cue, seeking, reward). This was performed for each of the blocked recording sessions (sucrose, 10% EtOH, 20% EtOH). These results are shown in Table 1. Unsurprisingly, there was significant heterogeneity, even within outcome category: during sucrose sessions neurons exhibited 15 different response profiles including significant responding during at least one epoch, during 10% EtOH sessions neurons exhibited 10 different profiles, and during 20% EtOH, neurons exhibited 13 different profiles. In all cases, the overall patterns seen in Figures 2, 3 were maintained (primarily inhibition during sucrose reward, etc.). However, response patterns diverged across epochs, virtually tiling potential pattern space. Of interest is the fact that neuronal responses during 20% self-administration were largely selective for one epoch and did not exhibit complex excitation/inhibition dynamics as observed during sucrose and 10% EtOH seeking. These results suggest that, rather than reporting a single signal during reward seeking, OFC neurons participate in different populations, each encoding different subcomponents of cue-evoked reward seeking.

**Table 1:** Distribution of response profiles across neurons during blocked sucrose, 10% EtOH, and 20% EtOH recording sessions

Sucrose				10% EtOH				20% EtOH			
Cue	Seeking	Reward	# Ns	Cue	Seeking	Reward	# Ns	Cue	Seeking	Reward	# Ns
0	1	–1	43	0	0	–1	37	–1	0	0	20
0	0	–1	30	–1	1	0	17	0	0	–1	11
–1	–1	–1	20	0	1	–1	7	1	0	0	10
0	–1	–1	16	–1	0	–1	5	0	0	1	9
0	1	0	12	–1	–1	0	4	0	1	0	9
1	0	0	8	–1	1	–1	2	1	1	1	8
–1	0	1	4	–1	0	0	1	0	1	1	5
–1	1	–1	4	0	–1	–1	1	0	–1	1	4
–1	0	–1	3	1	0	0	1	1	0	–1	3
–1	1	0	2	1	1	0	1	–1	0	–1	2
0	–1	0	2					1	0	1	2
–1	0	0	1					–1	–1	1	1
0	0	1	1					1	–1	1	1
0	1	1	1								
1	0	–1	1								
0	0	0	14	0	0	0	18	0	0	0	50

Neuron populations are ordered from those containing most to least members. Category of response to cue, seeking, or reward is demarcated by −1 (significant inhibition during that epoch), +1 (significant excitation during that epoch), or 0 (no significant activity during that epoch). At the bottom of each section is the number of non-responsive neurons [0,0,0]. Any combination of responses not shown in this table was not observed during recording.

We also measured changes in baseline activity to determine whether differences across and within blocked session outcomes were reflected in basal OFC firing rate. We found no significant differences in baseline activity (mean firing rate −8 to −4 s before trial start) across different sessions (i.e., sucrose vs 10% EtOH vs 20% EtOH: *F*_(2,435)_ = 0.65, *p* = 0.53, ANOVA). We further measured changes in baseline activity across individual sessions for each neuron to determine whether basal activity changed as a function of time, behavior, reward accumulation, etc. Mean pretrial baseline activity (mean firing rate −8 to −4 s before trial start) across each session was grouped into four epochs and comparisons were made across epochs. There were no significant differences across epochs in sucrose (*F*_(2.597,418.1)_ = 1.65, *p* = 0.18, repeated measures ANOVA), 10% EtOH (*F*_(2.075,230.3)_ = 2.672, *p* = 0.07), or 20% EtOH (*F*_(2.807,457.6)_ = 1.02, *p* = 0.38). Similar lack of effects was observed if other baseline epochs were analyzed. Together, these data indicate that OFC activity was stable both within sessions and that there were no significant differences in baseline OFC activity across sessions.

As noted in Materials and Methods, we stereotaxically targeted separate electrode bundles to mOFC and lOFC. Although our histologic analysis reliably demonstrated that all recording electrodes were located in OFC, we were not completely confident in our ability to analyze differences in the activity of mOFC versus lOFC neurons based on matching recording channels to histologic reconstruction of electrode placements (Fig. 2*A*). However, as a rough analysis we separated our recordings into putative mOFC versus lOFC based on stereotaxic placements of electrode arrays, independent of lesion sites, which spanned lOFC, vOFC, and mOFC. Based on this grouping, we found no significant differences in mOFC versus lOFC response indices during sucrose or 10% EtOH sessions, nor during cue or seeking epochs during 20% EtOH sessions (no main effect of mOFC vs lOFC or interaction effect; all *p* > 0.05; two-factor ANOVA), although there were significant main effects of outcome in both epochs, in line with the overall findings reported above. During the reward consumption epoch, we found a main effect of mOFC versus lOFC (*F*_(1,385)_ = 4.97, *p* = 0.03) and a main effect of outcome (*F*_(2,385)_ = 13.55, *p* < 0.0001), although no significant interaction effect. *Post hoc* analyses did not reveal clear effects of medial versus lateral in any specific condition (for example, mOFC vs lOFC during 20% EtOH). This overall effect appears to be driven primarily by stronger inhibition in mOFC versus lOFC across conditions (sucrose indices, mOFC: −0.17, lOFC: −0.13; 10% EtOH indices, mOFC: −0.13, lOFC: −0.12; 20% EtOH indices, mOFC: −0.09, lOFC: −0.03). These data are intriguing in the light of previous reports of differential value/preference coding by mOFC versus lOFC neurons (Burton et al., 2014; Lopatina et al., 2016). However, we stress that any conclusions based on these data are tentative because of incomplete histologic confirmation.

### Differential OFC signaling during consumption was not related to differential lick behavior

One potential explanation for the striking differential OFC activity observed during is that, rather than differentially encoding outcome, OFC activity was related to licking behavior. Since reward consumption was shorter for EtOH, particularly 20% EtOH trials compared with sucrose trials an argument might be that less OFC inhibition during 20% EtOH trials was because of shorter consumption epochs. Although this was largely accounted for by performing analyses on trial-by-trial consumption epochs, we performed a number of analyses to verify our conclusions. First, we correlated the trial-to-trial activity index of each neuron with lick duration to determine whether activity scaled with lick duration, using normalized response indices. We found that 11/162 neurons in sucrose, 6/171 neurons in 20% EtOH, and 6/113 neurons in 10% EtOH exhibited significant (*p* < 0.05) correlation with lick duration, indicating that there were minimal influences of lick duration on neural activity. Second, we combined all activity of all neurons in all trials and performed a total correlation between index and duration. We found significant correlations in all of these cases (sucrose: ρ = 0.09, *p* < 0.0001; 10% EtOH: ρ = −0.06, *p* = 0.029; 20% EtOH: ρ = −0.11, *p* < 0.0001). We were skeptical of the relevance of these results because of (1) the fact that sucrose and EtOH conditions had opposing correlation directions, suggesting no real OFC relationship with lick duration and (2) the very small ρ values, suggesting that statistical significance resulted from correlations performed on very large numbers (>1000 per analysis). Third, to assess whether this effect was driven by large numbers of data points, we calculated correlations for each condition on an animal-by-animal basis, reasoning that if this effect was valid, we should see it in each animal. Across sucrose recordings, we found significant (*p* < 0.05) correlations between lick duration and OFC activity in 2/16 rats. Across 10% EtOH sessions, we found significant correlations in 2/11 rats. Across 20% EtOH sessions, we found significant correlations in 1/12 rats. Two rats in 20% EtOH and one rat in 10% EtOH sessions consumed too little EtOH to permit analysis. We conclude from these findings that there was no relationship between reward consumption duration and degree of suppression of OFC activity. To confirm a lack of relationship between OFC activity and licking in this experiment, we plotted histograms time-locked to licks and lick bouts to determine whether there were peaks in activity as have been observed in previous studies in OFC (Gutierrez et al., 2010) and medial prefrontal cortex (Amarante et al., 2017). We observed no clear time-locked activity and did not pursue further analysis.

Another, related, possible alternate interpretation of differences observed during consumption is that, after the initiation of licking, there is a temporally consistent process of inhibition that occurs for all licking behavior independent of reward, that is truncated by shorter EtOH licking times thereby precluding observation of significant suppression during 20% EtOH consumption. To address this issue, we performed a sliding window analysis using 100-ms bins to determine when OFC firing was significantly inhibited during consumption relative to baseline. We performed this analysis on sucrose and 20% EtOH sessions, as these were the extremes with respect to reward consumption duration and because sucrose responses were strongly down-modulated and 20% EtOH responses were not. We calculated, on a neuron-by-neuron basis, the timestamps of the first of two consecutive 100-ms bins following the initiation of consumption that reached statistically significant inhibition compared with baseline. We then compared the relationship of this onset of inhibition to lick durations in both sucrose and EtOH. In sucrose sessions, the median latency for inhibition to develop postlick was 0.5 s. This was not different from that seen in the small number of neurons in which inhibition was observed in 20% EtOH trials (median = 0.7 s; *p* = 0.29, Wilcoxon rank sum). In contrast, the latency of inhibition onset was significantly different, and much shorter than the duration of licking measured in these analyses (sucrose duration median 6.38 s vs 20% EtOH median 5.47 s, all comparisons of inhibition latency vs consumption duration *p* < 0.0001; note that durations are slightly different from those shown in Fig. 1 because of inclusion only of trials in which inhibition of activity was measured). Although there was a significant difference between sucrose and 20% EtOH consumption duration, this difference did not overlap with the onset of potential inhibition, as seen in sucrose trials and in the small number of significantly inhibited neurons in EtOH trials. Beyond statistical significance, the overall differences in magnitude (<1 s for onset of inhibition vs >3 s for duration of consumption) strongly indicate that there was ample consumption of 20% EtOH to permit possible inhibition of OFC activity, if it were to occur. Instead, we conclude that inhibition of OFC activity during sucrose consumption was not present in the majority of neurons recorded during 20% EtOH consumption, independent of duration of licking.

### 20% EtOH seeking was strongly associated with homecage EtOH preference

**Figure 4.**
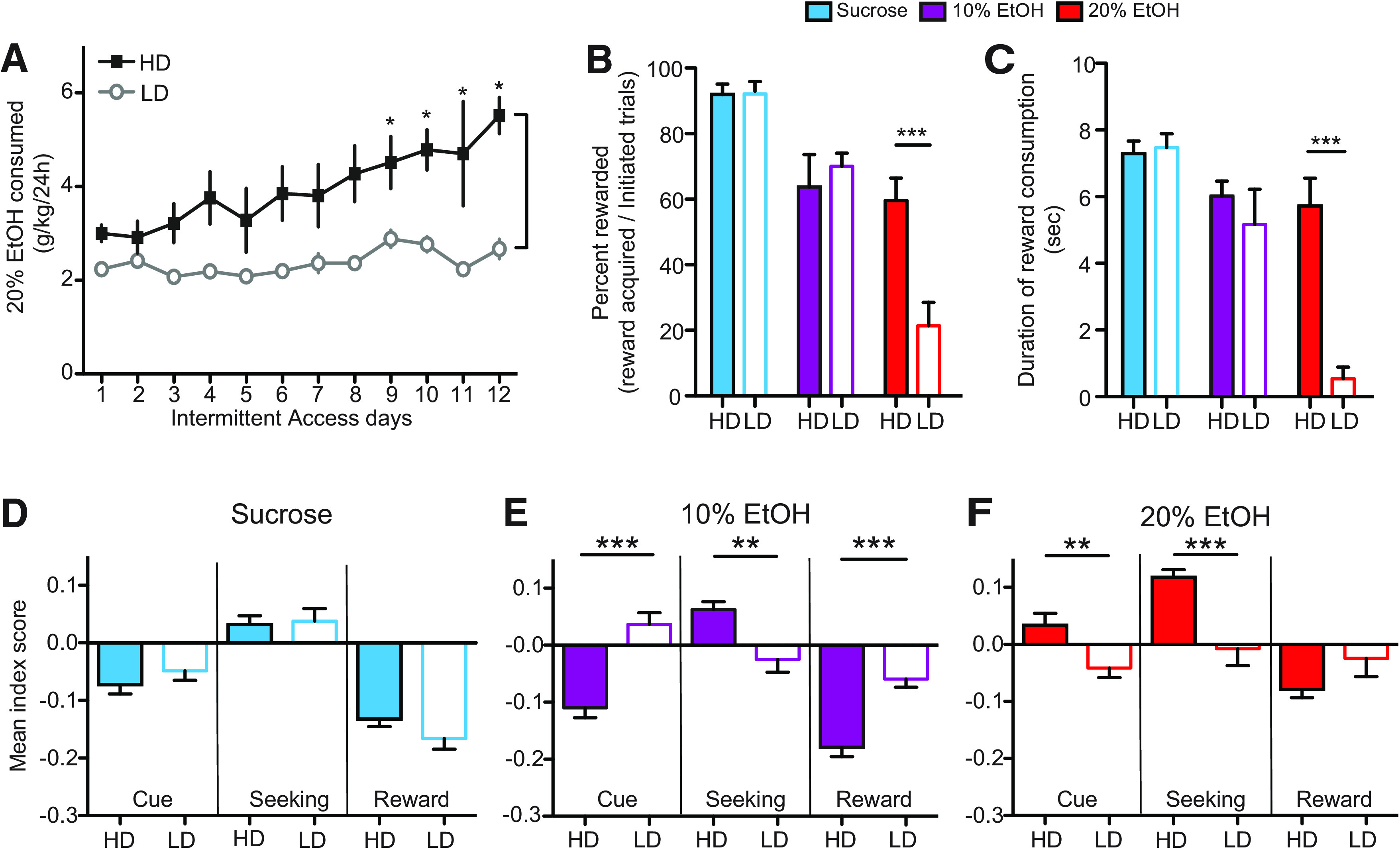
Rats were separated into HDs and LDs based on homecage EtOH consumption (see Materials and Methods). ***A***, HD rats exhibited significant escalation over the course of homecage intermittent access to EtOH whereas LD rats did not. ***B***, HD rats (filled bars) completed significantly more rewarded trials for 20% EtOH (red) than LD rats (open bars), but there were no differences for sucrose (blue) or 10% EtOH (purple) rewarded trials. ***C***, HD rats consumed significantly more 20% EtOH than LD rats, measured by lick duration, but there were no differences in consumption of sucrose or 10% EtOH. ***D***, OFC neuronal activity in HD versus LD rats was similar during sucrose cues, seeking, and consumption but was significantly different during cues, seeking, and consumption of 10% EtOH (***E***) and during cues and seeking of 20% EtOH (***F***). Overall strength of OFC signaling (either excitation or inhibition) was suppressed in LD rats relative to HD rats in EtOH sessions; *p *<* 0.05, **p *<* 0.01, ***p *<* 0.001.

Rats were separated into HDs and LDs based on average g/kg 20% EtOH consumed during homecage intermittent access (see Materials and Methods). Of the 16 rats studied in sucrose and 20% EtOH conditions, seven were classified as LD, and nine were classified as HD. Of the 12 rats studied in sucrose, 10% EtOH, and 20% EtOH conditions, five were classified as LD, and seven were classified as HD. HD rats escalated 20% EtOH consumption across intermittent access sessions, whereas LD rats did not (two-way ANOVA; main effect of day: *F*_(1,11)_ = 2.77, *p* = 0.002; main effect of HD/LD: *F*_(1,1)_ = 127.3, *p* *<* 0.0001; interaction *F*_(1,11)_ = 2.93, *p* = 0.001; Fig. 4*A*). Based on mean 24-h consumption in the last 3 d of homecage intermittent access, HD rats consumed significantly more EtOH than LD rats (HD: 5.0 ± 0.45 g/kg); LD: 2.35 ± 0.13 g/kg; *U* = 5 *p* *<* 0.0001, Mann–Whitney).

During operant testing, HD and LD rats exhibited significant differences in EtOH seeking and consumption (Fig. 4*B*,*C*). HD rats received significantly more 20% EtOH rewards than LD rats (*U* = 5, *p* *=* 0.004; Fig. 4*B*, red), but there were no differences in seeking for sucrose or 10% EtOH (sucrose: *U* = 31, *p* = 1; 10% EtOH: *U* = 14, *p* = 0.42). HD rats also consumed more 20% EtOH than LD, measured by the duration of licking (*U* = 3, *p* *=* 0.0006; Fig. 4*C*, red). There were no differences between HD and LD rats with respect to consumption of sucrose or 10% EtOH (sucrose: *U* = 29, *p* = 0.84; 10% EtOH: *U* = 20, *p* *=* 0.16). Average g/kg of 20% EtOH consumed on the final 3 d of intermittent access was also correlated with duration of reward consumption during each rewarded trial for 20% EtOH (*r* = 0.82, *p* *<* 0.0001) and 10% EtOH (*r* = 0.51, *p* *=* 0.007), but not sucrose: *r* = 0.15, *p* *=* 0.99).

### OFC activity during 10% and 20% EtOH seeking was associated with homecage EtOH preference

OFC neural activity was significantly different between HD and LD rats during EtOH seeking. There were no significant differences in OFC activity during sucrose cue presentation, reward seeking, and consumption in HD versus LD rats (cue: *U* = 3128, *p* = 0.71; seeking: *U* = 3126, *p* = 0.70; reward: *U* = 3010, *p* = 0.44, Mann–Whitney; Fig. 4*D*). In contrast, there were significant differences in OFC activity between HD versus LD rats during both 10% and 20% EtOH trials. OFC neurons in HD rats exhibited response profiles more similar to that seen during sucrose seeking (Fig. 4*E*,*F*), whereas OFC responses were significantly suppressed in LD rats. These differences were significant for all epochs in 10% EtOH sessions (cue: *U* = 639, *p* = 0.0007; seeking: *U* = 870, *p* = 0.0014; consumption: *U* = 416, *p* *<* 0.0001), and during cue and seeking epochs during 20% EtOH sessions (cue: *U* = 2204, *p* = 0.001; seeking: *U* = 1028, *p* = 0.0004; consumption: *U* = 1633, *p* = 0.44). Consumption differences during 20% EtOH sessions exhibited a similar HD/LD pattern as during 10% EtOH sessions, but were statistically underpowered during consumption for LD rats because of a limited number of 20% reward acquisitions by this population (Fig. 4*B*).

### OFC activity during sucrose and 20% EtOH interleaved trials was strongly associated with homecage EtOH preference

**Figure 5.**
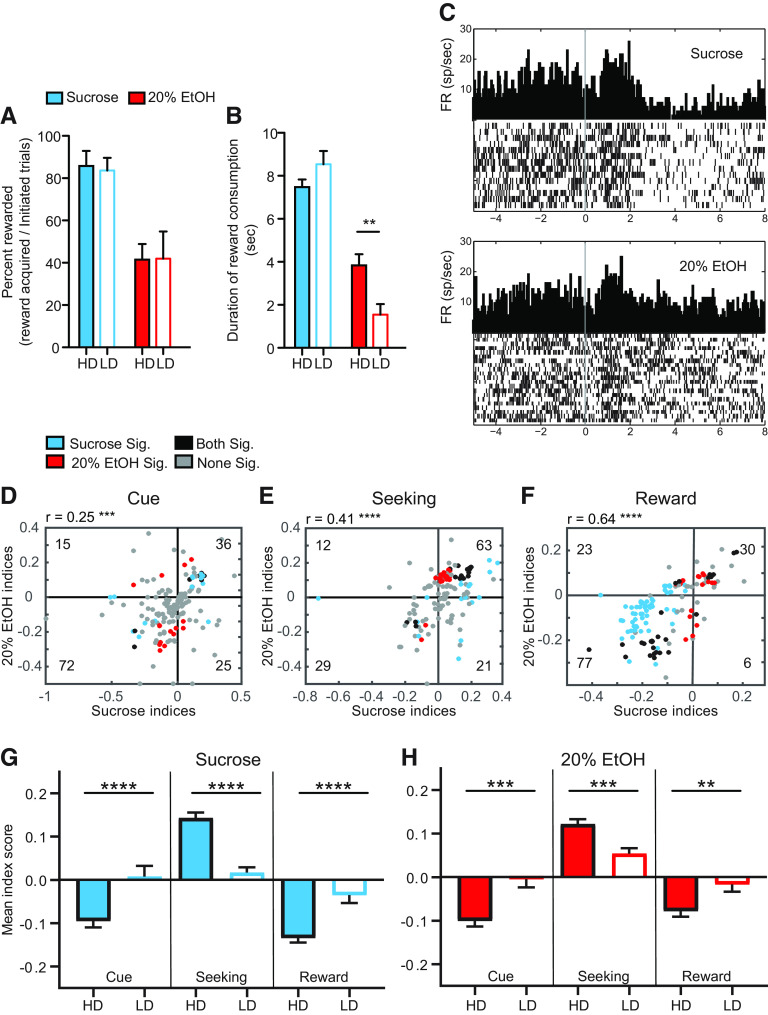
OFC neuronal activity was recorded during interleaved trials of 20% EtOH and sucrose. ***A***, During interleaved sessions, HD (filled bars) and LD (open bars) rats completed similar numbers of sucrose (blue) and 20% EtOH (red) trials. ***B***, However, LD rats consumed significantly less 20% EtOH than HD rats. ***C***, Example of activity from a single neuron in sucrose (top) and 20% EtOH (bottom) conditions. ***D–F***, Sucrose/EtOH index profiles for individual neurons recorded during interleaved trials of sucrose and 20% EtOH seeking. Recording the same neuron in both conditions allowed characterization of sucrose/EtOH index profiles for each neuron. Indices were calculated as in blocked conditions. Each dot represents sucrose/20% EtOH index combination for each neuron during cue (***D***, *n* = 156 neurons), seeking (***E***, *n* = 137 neurons), and reward (***F***, *n* = 137 neurons). Only neurons in which seeking and consumption trials were performed for both sucrose and 20% EtOH were included in seeking and reward plots; *r* and *p* values indicate significant correlations across all neurons in sucrose and EtOH trials, despite a bias toward significant selectivity in encoding of one versus another outcome. Blue, red, black, and gray dots represent neurons significantly activated/inhibited during sucrose conditions only, 20% EtOH only, both, and neither condition, respectively. Although a subset of neurons exhibited significant modulation in both sucrose and EtOH trials (black dots: 8 in response to cue, 17 during seeking, and 29 during consumption), the majority of significantly-influenced neurons exhibited selectivity for sucrose (blue dots: 14 cue, 16 seeking, and 58 consumption) or EtOH (red dots: 15 cue, 22 seeking, and 19 consumption). Numbers in each quadrant indicate numbers of neurons falling within that quadrant. Neurons with an index of 0 in one axis were not counted in quadrant totals. Most neurons showed inhibition during sucrose and EtOH cues, excitation during sucrose and EtOH seeking, and inhibition during sucrose and EtOH consumption, in line with patterns observed during blocked conditions. ***G***, ***H***, OFC activity was similar in sucrose (***G***) and 20% EtOH (***H***) trials in HD rats, but activity of OFC neurons in LD rats was again suppressed relative to activity of those in HD rats. This was true for neuronal responses to the cue, during reward seeking, and during sucrose/EtOH consumption; **p *<* 0.01, ***p *<* 0.001, ****p *<* 0.0001.

In order to characterize how the same OFC neurons fired during sucrose and EtOH seeking, we also recorded OFC activity during sessions in which sucrose and 20% EtOH trials were pseudorandomly interleaved (Materials and Methods). During interleaved sessions HD and LD rats exhibited similar proportions of sucrose and 20% EtOH rewarded trials, and similar duration of sucrose consumption (*p* > 0.05; Fig. 5*A*,*B*), but LD rats consumed significantly less EtOH measured by duration of consumption (*U* = 9, *p* *=* 0.007; Fig. 5*B*)

OFC activity was significantly changed during cue, seeking, and reward consumption epochs. Figure 5*C* shows activity of an example neuron recorded in sucrose and EtOH trials. Across the population of OFC neurons, response indices were highly correlated in sucrose and EtOH trials (Fig. 5*D–F*). Neurons exhibited strong, significant biases toward inhibition during cue presentation (χ^2^(1) = 27.71, *p* *=* 0.0008; χ^2^), excitation during reward seeking (χ^2^(1) = 24.01, *p* *<* 0.0001) and inhibition during reward consumption (χ^2^(1) = 40.51, *p* *<* 0.0001), in line with responses recorded during blocked conditions. Although a subset of neurons exhibited significant modulation in both sucrose and EtOH trials, the majority of neurons with significant changes in firing rate exhibited selectivity for sucrose or EtOH. As with neurons recorded during the blocked conditions, we observed significant heterogeneity in response profiles across neurons (Table 2). Although the variability does appear to be greater during interleaved conditions, this is to some degree driven by the increased numbers of parameters used to classify neurons (six vs three) and there are large populations of neurons with specific types of encoding in one subcondition or the other (e.g., inhibition during sucrose consumption). Thus, although the overall OFC population treated sucrose and EtOH as points in a continuum of rewards, as evidenced by correlated activity, individual neurons exhibited a diverse array of epoch-specific response biases and selective activity for sucrose or EtOH.

**Table 2:** Distribution of response profiles across neurons during interleaved sucrose, 10% EtOH, and 20% EtOH recording sessions

Sucrose			20% EtOH			
Cue	Seeking	Reward	Cue	Seeking	Reward	# Ns
0	0	–1	0	0	0	28
0	0	–1	0	1	0	10
0	0	–1	0	0	–1	9
0	1	–1	0	0	0	8
0	0	0	0	1	1	4
1	1	1	1	1	1	4
0	0	–1	–1	1	0	3
0	1	–1	0	0	–1	3
0	1	0	0	1	0	3
–1	0	–1	–1	0	0	2
–1	0	–1	0	0	–1	2
0	0	–1	–1	0	–1	2
0	0	–1	–1	0	0	2
0	0	–1	0	1	1	2
0	0	1	0	0	1	2
0	1	–1	0	1	0	2
1	0	0	0	0	1	2
1	0	0	1	0	1	2
1	1	0	1	1	1	2
–1	0	–1	–1	0	–1	1
–1	0	0	–1	0	0	1
–1	0	0	0	0	0	1
–1	0	0	0	0	1	1
0	–1	–1	–1	–1	0	1
0	–1	–1	–1	0	0	1
0	–1	–1	0	0	–1	1
0	–1	0	0	–1	–1	1
0	–1	0	0	0	–1	1
0	–1	0	1	–1	–1	1
0	–1	1	0	–1	–1	1
0	0	0	–1	0	0	1
0	0	0	0	–1	–1	1
0	0	0	0	0	–1	1
0	0	0	0	0	1	1
0	0	0	0	1	0	1
0	1	–1	0	1	1	1
0	1	0	0	1	1	1
0	1	0	1	0	0	1
1	0	0	0	0	0	1
1	1	0	0	0	0	1
1	1	0	0	1	1	1
1	1	1	0	1	1	1
						
0	0	0	0	0	0	19

Format is the same as Table 1, except that the same neurons were recorded in sucrose/20% EtOH interleaved sessions and neurons are categorized based on responses in both trial types.

OFC activity during interleaved trials was strikingly different in HD versus LD rats (Fig. 5*G*,*H*). In HD rats, OFC activity during sucrose and 20% EtOH trials followed a similar pattern: inhibition during cue presentation, excitation during seeking, and inhibition during consumption, similar to that seen during blocked sessions. In contrast, LD rat OFC neurons exhibited suppressed responses during all epochs. During cue presentation there was a significant main effect of preference (*F*_(1,135)_ = 20.51, *p* *<* 0.0001; two-way mixed ANOVA). During the seeking epoch, there was a significant main effect of preference (*F*_(1,135)_ = 40.99, *p* *<* 0.0001) and significant interaction effect between preference and reward type (*F*_(1,135)_ = 13.41, *p* = 0.0003). Finally, during the reward consumption epoch, there was a significant main effect of preference (*F*_(1,135)_ = 19.73, *p* *<* 0.0001), a significant main effect of reward type (*F*_(1,135)_ = 17.56, *p* *<* 0.0001), and a significant interaction effect (*F*_(1,135)_ = 4.61, *p* = 0.0336). These results demonstrate a clear relationship between EtOH preference and OFC activity in HD versus LD rats. OFC neurons in HD rats responded more similarly for sucrose and EtOH, encoding both as palatable rewards. In contrast, OFC neurons in LD rats responded weakly for both sucrose and EtOH, suggesting a possible suppressive effect of the presence of EtOH during interleaved sessions.

## Discussion

Here, we demonstrated that OFC neuronal activity encodes individual preferences for alcohol. OFC activity was significantly increased or decreased during both sucrose and alcohol seeking, but the features of their alcohol-associated responses were directly related to alcohol palatability and homecage drinking. OFC neuronal activity was most strongly affected in sucrose trials, followed by 10% EtOH and 20% EtOH, in line with behavioral preferences. Furthermore, OFC neurons in HD rats exhibited stronger responses during EtOH seeking than in LD rats, supporting the hypothesis that individual differences in relative preference for natural and drug rewards are encoded in OFC (Tremblay and Schultz, 1999; Padoa-Schioppa and Assad, 2006; Schultz, 2010; Guillem and Ahmed, 2018b; Guillem et al., 2018) and demonstrating this in the context of alcohol seeking. Importantly, differential OFC activity across individuals and outcomes cannot be explained exclusively by behavioral measures such as mechanics of reward consumption. Analysis of consumption-associated activity was based on periods of time when the rats were consuming sucrose or EtOH, meaning that consumption-associated differences in activity across outcomes or individuals were not influenced by periods of non-consumption. Along these lines, differential OFC activity could not be explained by individual variation in consumption duration, since, in some cases, rats with equivalent consumption behaviors exhibited significantly different OFC neuronal responses [e.g., 10% EtOH in HD vs LD (Fig. 4) and sucrose in HD vs LD (Fig. 5)]. This was formally supported by statistical analysis showing very little correlation between OFC activity and reward consumption duration. Our results indicate that OFC function is fundamentally different in high versus low alcohol-preferring individuals. They further suggest that OFC neurons in alcohol use disorder-prone or -diagnosed individuals may respond more robustly to alcohol and alcohol cues, conferring enhanced value and driving enhanced alcohol motivated behavior. Although this is somewhat speculative based on our current results, our data, along with human research to date, suggest that OFC should continue to be investigated in this context.

Our data are well aligned with previous work. In humans, OFC is activated during alcohol craving (Myrick et al., 2004, 2008; Lukas et al., 2013; Schacht et al., 2013a,b, 2014), and endgenous opioid release is induced by alcohol consumption in heavy drinkers (Mitchell et al., 2012). Inactivation of OFC in mice exposed to chronic EtOH vapor increased consumption of quinine-adulterated EtOH (den Hartog et al., 2016), and lOFC lesions increased alcohol consumption in rats (Ray et al., 2018), both of which suggest a regulatory role for OFC in alcohol use. This is supported by reports that chronic EtOH disrupts goal-directed behavior and suppresses OFC firing *in vitro* and that DREADD activation of OFC activity restores goal-directed behavior (Renteria et al., 2018). OFC inactivation in rats decreases cued or context-driven reinstatement of EtOH seeking, arguing that OFC activity may facilitate EtOH seeking (Bianchi et al., 2018). These latter results indicate that OFC contributes to EtOH seeking, in line with preference-associated differences observed here. Whether increased OFC activity induces or suppresses alcohol seeking may depend on a number of factors such as species, withdrawal state, and OFC subregion.

There is also an impact of acute and chronic EtOH on structure and function of OFC neurons. Chronic EtOH increased spine density in OFC neurons (McGuier et al., 2015; but see Holmes et al., 2012; DePoy et al., 2013). Acute EtOH decreased (Badanich et al., 2013a) and chronic EtOH exposure increased (Nimitvilai et al., 2016, 2017a) or decreased (Nimitvilai et al., 2017b; Renteria et al., 2018) OFC neuronal excitability. As with behavioral studies, there is some variability across species and paradigms, but there is a clear influence of EtOH on OFC structure and function, in line with the behavioral physiological results reported here.

OFC neuronal activity tracked individual preference, in some cases independent of operant behavior. During blocked 10% EtOH trials, HD and LD rats exhibited no significant differences in EtOH seeking or consumption (Fig. 4*B*,*C*). However, OFC activity was significantly different in HD versus LD rats in these trials (Fig. 4*E*). OFC neurons fired more strongly in HD rats, and the patterns of responses in HD rats were similar in sucrose and 10% EtOH conditions (Fig. 4*D*,*E*). This discontinuity between OFC activity and behavior demonstrates a stronger role in encoding alcohol preference versus seeking behavior. At the same time, the effects of preference on OFC activity during 20% EtOH seeking mapped clearly onto behavior. HD rats were more highly motivated than LD rats during both blocked and interleaved 20% EtOH trials (Figs. 4*B*,*C*, 5*B*), and OFC activity changes were stronger in HD than LD rats during these sessions (Figs. 4*F*, 5*H*). During interleaved sessions, OFC activity was also suppressed in LD rats during sucrose trials (Fig. 5*G*), again in contrast with no differences in sucrose seeking behavior (Fig. 5*A*). One possible explanation may be that subjective value of sucrose may have been compromised for LD rats by the presence of EtOH trials during sucrose sessions, although other explanations are possible as well. Intriguingly, if this is true, this indicates that OFC activity was different across individuals (suppressed in LD rats) although behavior was consistent, thereby potentially dissociating OFC encoding of preference/value (here demonstrated by homecage alcohol preference) from behavior, potentially driven by alternate brain systems. Although speculative given the present results, these data point to an important future study investigating potential dissociations between behavioral versus neural representations of preference or value.

We note a number of conceptual limitations in this study. We were interested in how OFC neuronal activity differed based on differences in motivation to consume EtOH. We use the term individual preference as a measure of willingness to intake alcohol, measured behaviorally, but we note that preference is frequently characterized by measurements such as comparing EtOH versus water intake. Because we did not perform simultaneous two-bottle EtOH versus water measurements, we cannot conclusively say that the differences in alcohol consumption are preference, per se, but we note that individual variability in alcohol intake, including variability that arises over time during intermittent homecage access, is typically concurrent with alcohol preference as measured in two-bottle tests. We also note that individual differences in OFC activity (or differences in HD vs LD rats) may have been driven by differences in overall EtOH intake history. This fascinating question of innate versus exposure-driven differences in EtOH motivation is something that we are currently exploring. We also acknowledge that levels of drinking performed by high drinking rats here are lower than that observed using other techniques such as high-drinking rat strains, or chronic intermittent exposure to EtOH vapor. Although high drinking rats frequently consumed high levels of EtOH (in some cases over 10 g/kg/24 h), and were, on average, in line with previous reports of high drinking in outbred rats (Simms et al., 2008; George et al., 2012; Carnicella et al., 2014; Momeni and Roman, 2014; Spoelder et al., 2015), we cannot say for sure that drinking in this study was driven by pharmacological elements of EtOH as opposed to taste or palatability aspects, particularly given that we did not measure blood EtOH levels. Given the role of the OFC in processing taste and taste preferences (Rolls, 2015), this issue is something that should be addressed in future studies.

Together, these results support the hypothesis that OFC encodes outcome preference or value. The data are aligned with models of inferred value representation (Jones et al., 2012; Baltz et al., 2018; Sadacca et al., 2018). Neural responses to sucrose were suppressed in LD rats by the possibility of receiving 20% EtOH in interleaved sessions, potentially indicating an updating of cue/outcome representation when the less-preferred alcohol was an option. However, our data also support the representation of cached value in OFC activity. These preference-associated signals were most strongly associated with homecage drinking-driven alcohol preference independent of operant alcohol seeking behaviors. These potentially conflicting representations may derive from the effect of alcohol history on HD versus LD rats. In HD rats, a history of EtOH exposure may have produced a more rigid OFC representation of cached value and habitual behavior, resulting in similar encoding of sucrose and EtOH and overall greater motivation for EtOH. This is directly in line with recent results demonstrating chronic alcohol-induced disruption of OFC-dependent goal-directed behavior (Renteria et al., 2018). In contrast, LD rats maintain flexible behavior (e.g., sucrose consumption, alcohol avoidance) due in part to changes in OFC activity depending on the outcome. More work needs to be done to fully explain the nature of alcohol preference encoding, such as including other types of outcomes (water, aversive outcomes such as quinine, etc.). Furthermore, understanding cause and effect will be of substantial future importance, e.g., do HD rats have different OFC activity than LD rats because of some genetic or developmental feature or is it a consequence of high levels of alcohol drinking? Similar issues have been raised in studies of dopamine neuron encoding of individual alcohol preference in mice (Juarez et al., 2017).

An additional question for future work is the degree to which differential outcome encoding is reflected during cue presentations, actions, or acquisition of the outcome itself. Many previous studies have shown OFC responses to cues or actions associated with different outcomes, that vary depending on the outcome itself (Stalnaker et al., 2015; Sharpe and Schoenbaum, 2016). In some cases, this may related to preference (Tremblay and Schultz, 1999; Padoa-Schioppa and Assad, 2006; Schultz, 2010; Guillem and Ahmed, 2018b; Guillem et al., 2018) or, in others, it may relate to outcome identity (McDannald et al., 2014b). This is of interest in the context of the current findings, in which some differences were selective excitatory responses (during seeking) whereas others were inhibitory (during consumption). One possible explanation that demands further investigation is that different populations of OFC neurons encode cues versus actions versus outcomes themselves and that dynamics (relationship of excitation to inhibition) vary at each stage of the behavior. This hypothesis is supported by the observed heterogeneity in the neuronal populations recorded here (Tables 1, 2).

Based on these and related data, it is clear that a neural circuit framework including multiple neural networks should be considered to address these issues. As demonstrated by our work and others, characterization of individual variability in neural circuit representation of alcohol preference has relevance to a better understanding of the neural basis of alcohol use and potentially even how differential alcohol use contributes to alcohol use disorder.

## Synthesis

Reviewing Editor: Mark Laubach, American University Decisions are customarily a result of the Reviewing Editor and the peer reviewers coming together and discussing their recommendations until a consensus is reached. When revisions are invited, a fact-based synthesis statement explaining their decision and outlining what is needed to prepare a revision will be listed below. The following reviewer(s) agreed to reveal their identity: Matthew Roesch. Note: If this manuscript was transferred from JNeurosci and a decision was made to accept the manuscript without peer review, a brief statement to this effect will instead be what is listed below.

Dear authors,

Please address the comments from Reviewer 2 in your revised manuscript. I am also including comments from Reviewer 1, who saw the paper at JN. They recommended accepting as is. If you make any changes based on their comments, please be sure to note them in your summary of changes. I would strongly suggest that you try to address the first two points raised by Reviewer 1, and also consider plotting spikes around licks during the reward period. Studies from my own lab have found evidence for spike entrainment to licking (Amarante et al., JN, 2017), and it would be interesting to know if these findings, made in a free access procedure, translate to your design. That said, your recordings are ventral to where my own group has worked, so you might not find entrainment. (We think that entrainment is due to cross-area connections in the more dorsomedial cortex given the close proximity to oral and facial motor cortex in the adjacent frontal agranular cortex.)

-Mark

Reviewer 1

In my opinion the paper is much improved and acceptable for publication in eNeuro in its current format. The concerns mentioned below are picky and not necessary to be addressed for publication.

1. I was not completely satisfied by the response that self-initiated aspects of the task allowed for complete segregation of trial epochs. There is still bleed over between epochs (unless I am missing something). For example, looking at figure 2C, the cue epoch is 1 s plus 100 ms so that encompasses the cue time, seeking, and the beginning of reward. Likewise, the reward seeking epoch starts 400 ms before reward, which is nearly half the cue epoch. Overall the epoch analysis captures important features of the average population histogram so I’m ok with it.

2. I still don’t think that the authors can completely rule out that activity reflects the vigor that rats licked for reward. One argument against this interpretation was that rats licked for 20% ethanol and there were no decreases, but they probably are not licking much for 20% and, on average, only appear to lick for about 3-4 seconds. The same problem exists when comparing sucrose to 10% ethanol. It is also argued that this could not explain cue activity but I’m sure reaction times differed between the two groups and the cue epoch encompasses seeking and part of reward. Finally, the authors suggest the declines in firing can’t reflect licking because both LD and HD rats held for reward consumption, but showed different firing. Again, the problem I had with this argument is the LD probably licked less vigorously and licked far less (∼1 s)

To fully address this issue, authors should consider plotting activity based on lick time for each trial type and group. Also they could see if there are correlations between firing rate and licking.

Ultimately how much rats lick and how much they value reward go hand in hand so it is hard to dissociate the two, thus I’m ok with it for eNeuro.

3. Lastly, I agree with the authors that they used the appropriate statistics to determine shifts from zero for their distributions, but I also think they need to compare distributions against each other. This is probably most important for figure 3. For example, just because one distribution is shifted but the other one is not does not mean the distributions are different from each other.

Reviewer 2

The present manuscript focuses on data from in vivo recording studies performed in outbred rat OFC during alcohol self-administration. OFC structure and function has been shown altered in addicts, as well as animal models of alcohol use and dependence. In the decision-making world, OFC historically has been implicated in value processing, palatability, representing associative information. Here, the authors report findings data that suggest OFC representation of cue-signaled seeking and consumption (nicely limited to periods of licking) of alcohol, is somewhat different than that of sucrose, and that rats, who consumed more alcohol in a home-cage setting and showed different OFC representation than rats who consumed less alcohol in the home cage. So, OFC neurons are modulated more to cues and outcome that support more seeking and consumption behaviors. However, statistical comparisons on neural activity data are not made across substances, limiting direct comparisons and interpretations. Much of this data is by comparing recorded populations on a sucrose day and on an ethanol day, although there are trials where sucrose and ethanol self-administration is interwoven allowing for comparison of the same neuron during both types of SA. This data could offer support for one hypothesis that alcohol exposure changes OFC function, or another hypothesis that genetic variability between subjects could lead a portion to drink more alcohol and have different representation of alcohol-related information. The data does show that in general, OFC activity changes during cue-signaled alcohol seeking and consumption in a way that differs somewhat from sucrose. The authors responded appropriately to prior reviewers. Although there are some definitions and words usage that is not supported, the overall findings are novel. Some additional analyses may serve to strengthen their claims.

The use of the word preference is misleading. I kept waiting for a preference assessment in the home-cage drinking set up where high and low drinkers were split that was never performed. What was done, was examining total 24 h consumption across training and taking a split previously used in the literature. This is not a preference, but a different consumption history, with an arbitrary split. This is a phenotype of slighter higher drinking. Even high drinking in this study is misleading, as rats are barely drinking over a 24 h period. No BECs are reported, and without that, whether the low overall consumption measured would be pharmacologically relevant at any point in a daily cycle is not clear. I appreciate the goal of looking at individual differences, but without more evidence, the claim of high drinkers or higher preference is not supported. Different consumption histories, and total amount of alcohol consumed are different. What is a more likely contributor, is differences in palatability and the ability for experience to enhance any palatability change, and given the use of Long Evans, a reduction in aversion to taste properties of alcohol seems likely. The authors do mention palatability, but there is quite an extensive and literature of OFC and palatability. Whether OFC is directly involved in determining palatability, or represents aspects of palatability and its use may be of interest for alcohol self-administration.

EDITOR’S NOTE: Please revise the text to address this issue of preference and palatability. Please include any preference data, e.g. from home cage testing, if you have it. This is a major issue to focus on for the revised manuscript.

Missing is some reporting on the dynamics observed within a neuron. This is a dynamic task. The activity of neurons is split up by epochs and the average reported, whether it is increasing or decreasing from baseline, and how many neurons showed similar effects. This is then used to build a picture of what “OFC” activity looks like. This is fairly standard for the field, but almost a bit misleading and the take away picture is not clear. Figure 2D was per animal, but I don’t think Figure 3 was. Grouping them this way can be misleading as to what the overall output of OFC may look like. Careful phrasing can reduce this, just saw more down modulation, or more up modulation etc.

EDITOR’S NOTE: I agree that it is hard to judge dynamics as well as variability over neurons, given how you have shown the data. Having one raster is much appreciated, but how does the ensemble average capture the diversity of responses over neurons and the epochs shown in Fig 2D? As you recorded from multiple neurons simultaneously, could you include a new figure showing activity across one or two sets of simultaneously recorded neurons to depict cross neuron variability and temporal dynamics? (This could be made quickly using a program such as NEx.)

Outcome signaling or reward signaling is thought of as the ground truth for OFC activity. But here, most neurons that shown modulation are down modulated. Do the authors think of cue or seeking as outcome signaling?

EDITOR’S NOTE: Please revise the text to address this issue.

One thing that did stand out, was the greater down modulation during licking behaviors, at least in the sucrose and the 10% ethanol, less so for 20%. Given that activity analysis was limited to licking periods, and rats drinking 20% licked less and for shorter periods, is OFC activity steady across the period?

EDITOR’S NOTE: Please revise the text to address this issue. You might want to use a sliding window measure of firing rate over the period of reward to address this issue.

One of the section titles seems to be misleading; was there an analysis that showed home cage ethanol preference predicted ethanol seeking? That would be a correlation? Group differences are shown, but we don’t know about prediction.

EDITOR’S NOTE: Please revise the text to address this issue.

In this frame work, simple correlations between total change in modulation predict longer bout sizes or more licks?

EDITOR’S NOTE: Please revise the text to address this issue. I would like to suggest that you use Spearman rank correlation to examine relationships between firing rate and bout duration + lick count per trial.

Some care should be taken with wording. For example, OFC neurons treating ethanol and sucrose as less palatable in the interleaved trials in LD rats (last sentence of results and in discussion as well). I don’t believe there is supporting data for that claim; as interleaved trials could exert a lot of other effects other than an anticipatory contrast effect on consumption. Even that, isn’t necessarily tied to palatability.

EDITOR’S NOTE: Please revise the text to address this issue.

In the discussion, newly added wording about cue activity differing not explained by behavior because there was similar nose-poke demands, is not correct. Just because the rules were the same across conditions, does not mean the behavior emitted and the time course with which it is emitted are.

EDITOR’S NOTE: Please revise the text to address this issue.

## Author Response

Editor:

Please address the comments from Reviewer 2 in your revised manuscript. I am also including comments from Reviewer 1, who saw the paper at JN. They recommended accepting as is. If you make any changes based on their comments, please be sure to note them in your summary of changes. I would strongly suggest that you try to address the first two points raised by Reviewer 1, and also consider plotting spikes around licks during the reward period. Studies from my own lab have found evidence for spike entrainment to licking (Amarante et al., JN, 2017), and it would be interesting to know if these findings, made in a free access procedure, translate to your design. That said, your recordings are ventral to where my own group has worked, so you might not find entrainment. (We think that entrainment is due to cross-area connections in the more dorsomedial cortex given the close proximity to oral and facial motor cortex in the adjacent frontal agranular cortex.)

Thank you for the opportunity to submit a revised manuscript. We have gone through and addressed all of the reviewer comments, including those from Reviewer 1, described below. All edits to the text are shown in blue font.

We plotted spikes around licks and lick burst onsets and found no noticeable lick-synchronized responses, definitely nothing like what was shown in mPFC (Amarante et al. 2017) or in a previous study of the OFC (Gutierrez et al., 2010). Given that a major finding of our paper related to reward consumption is a pronounced suppression of activity, this is not surprising, but it would have been interesting to see global suppression with lick-burst-related spikes superimposed, so it was definitely worth a look. That lick bursts have been seen previously suggests to us that, at least in OFC, there might be some task dependence influence on lick-associated activity. However, having found no clear examples of this activity, we did not pursue analysis further. We did, however, perform four additional analyses to address the Reviewers questions about lick-related inhibition. These, and a comment about lick-burst activity, are now included in the revised manuscript.

Reviewer 1:

In my opinion the paper is much improved and acceptable for publication in eNeuro in its current format. The concerns mentioned below are picky and not necessary to be addressed for publication.

1. I was not completely satisfied by the response that self-initiated aspects of the task allowed for complete segregation of trial epochs. There is still bleed over between epochs (unless I am missing something). For example, looking at figure 2C, the cue epoch is 1 s plus 100 ms so that encompasses the cue time, seeking, and the beginning of reward. Likewise, the reward seeking epoch starts 400 ms before reward, which is nearly half the cue epoch. Overall the epoch analysis captures important features of the average population histogram so I’m ok with it.

Thank you for bringing this to our attention. There were some errors in the description of the task timing and analysis epochs. The correct epochs are as follow:

Task timing: 100 ms after nosepoke the tone turned on for 400-600 ms (independent of response). This is now correctly described in the revised manuscript.

The analysis epochs were as follow:

-Cue: cue+100ms.

-Seeking: 400 ms pre-first-lick

-Consumption: First rewarded lick to last rewarded lick

An analysis of behavior shows that the epochs chosen for analysis appropriately selected separate behaviors.

-In sucrose trials: median cue-exit RT = 110 ms, median exit-first lick RT = 890 ms.

-In 10% EtOH trials: median cue-exit RT = 210 ms, median exit-first lick RT = 960 ms.

-In 20% EtOh trials: median cue-exit RT = 170 ms, median exit-first lick RT = 1355 ms.

Based on behavioral responses, analysis epochs are well-matched with behavior. The Cue epoch captures the initial cue-evoked decision to initiate exiting the nosepoke, but does not capture reward well-entry, and the Seeking epoch captures behavior leading to well-entry, but not cue and/or nosepoke exit.

The “Cue” epoch was likely cue + initiation of response, but this is almost impossible to avoid in any behavioral task. Even when a post-cue prolonged hold epoch is required, a cue dictating a response will almost necessarily initiate programming the response behavior.

We hope that the reviewer is convinced that the epochs do not bleed into one another, and we appreciate the opportunity to clarify this. These additional measurements are now included in the revised manuscript.

2. I still don’t think that the authors can completely rule out that activity reflects the vigor that rats licked for reward. One argument against this interpretation was that rats licked for 20% ethanol and there were no decreases, but they probably are not licking much for 20% and, on average, only appear to lick for about 3-4 seconds. The same problem exists when comparing sucrose to 10% ethanol. It is also argued that this could not explain cue activity but I’m sure reaction times differed between the two groups and the cue epoch encompasses seeking and part of reward. Finally, the authors suggest the declines in firing can’t reflect licking because both LD and HD rats held for reward consumption, but showed different firing. Again, the problem I had with this argument is the LD probably licked less vigorously and licked far less (∼1 s)

To fully address this issue, authors should consider plotting activity based on lick time for each trial type and group. Also they could see if there are correlations between firing rate and licking.

Ultimately how much rats lick and how much they value reward go hand in hand so it is hard to dissociate the two, thus I’m ok with it for eNeuro.

We performed 5 analyses to address this issue of whether differences in consumption-related activity across outcomes could be simply explained by duration of licking in each session.

First, we determined whether single neurons exhibited lick-correlated activity on a short (msec) timescale as has been observed in (Amarante et al., 2017, Gutierrez et al., 2010). We found no instances of lick-correlated activity as defined by a significant peak around time 0.

Second, we correlated the trial-to-trial activity index of each neuron with lick duration to determine if activity scaled with lick duration, as suggested by the reviewer. We found that 11/162 neurons in sucrose, 6/171 neurons in 20% EtOH, and 6/113 neurons in 10% EtOH exhibited significant (p<0.05) correlation with lick duration, indicating to us that there were minimal influences of lick duration on neural activity.

Third, we combined all activity of all neurons in all trials and performed a total correlation between index and duration. We found significant correlations in all of these cases (sucrose: rho = 0.09, p = 1.15e-17; 10% EtOH: rho = -0.06, p = 0.029; 20% EtOH: rho = -0.11, p = 2.74e-4). We were skeptical of the relevance of these results due to a) the fact that sucrose and EtOH conditions had opposing correlation directions, suggesting no real OFC relationship with lick duration and b) the very small rho values, suggesting that statistical significance resulted from correlations performed on very large numbers (>1000 per analysis).

Fourth, to assess whether this effect was driven by large numbers of data points, we calculated correlations for each condition on an animal-by-animal basis, reasoning that if this effect was valid we should see it in each animal. Across sucrose recordings, we found significant (p<0.05) correlations between lick duration and OFC activity in 2/16 rats. Across 10% EtOH sessions, we found significant correlations in 2/11 rats. Across 20% EtOH sessions we found significant correlations in 1/14 rats. Two rats in 20% EtOH and one rat in 10% EtOH sessions consumed too little EtOH to permit analysis.

A fifth analysis, measuring the latency of onset of reward-consumption-evoked inhibition (and showing that it occurs well before the median endpoint of consumption of 20% EtOH) is described in response to Reviewer 2, below, and again indicates that duration of consumption was unrelated to the differential suppression of OFC activity during sucrose and ethanol consumption.

Our conclusion from all of these analyses is that, although there may be some lick-related signaling in OFC as reported previously, this is a very minor contributor to the significant differential suppressive effects observed across reward outcome and high/low drinking rats in this study. This does not negate the possibility that OFC neurons encode lick related behaviors in other contexts, but indicates that lick duration does not account for differences we observed across outcome conditions. These analyses and conclusions have been included in the revised manuscript.

3. Lastly, I agree with the authors that they used the appropriate statistics to determine shifts from zero for their distributions, but I also think they need to compare distributions against each other. This is probably most important for figure 3. For example, just because one distribution is shifted but the other one is not does not mean the distributions are different from each other.

We have now included the suggested analyses which show statistically significant differences among distributions. Thank you for the suggestion.

Reviewer 2:

The present manuscript focuses on data from in vivo recording studies performed in outbred rat OFC during alcohol self-administration. OFC structure and function has been shown altered in addicts, as well as animal models of alcohol use and dependence. In the decision-making world, OFC historically has been implicated in value processing, palatability, representing associative information. Here, the authors report findings data that suggest OFC representation of cue-signaled seeking and consumption (nicely limited to periods of licking) of alcohol, is somewhat different than that of sucrose, and that rats, who consumed more alcohol in a home-cage setting and showed different OFC representation than rats who consumed less alcohol in the home cage. So, OFC neurons are modulated more to cues and outcome that support more seeking and consumption behaviors. However, statistical comparisons on neural activity data are not made across substances, limiting direct comparisons and interpretations. Much of this data is by comparing recorded populations on a sucrose day and on an ethanol day, although there are trials where sucrose and ethanol self-administration is interwoven allowing for comparison of the same neuron during both types of SA. This data could offer support for one hypothesis that alcohol exposure changes OFC function, or another hypothesis that genetic variability between subjects could lead a portion to drink more alcohol and have different representation of alcohol-related information. The data does show that in general, OFC activity changes during cue-signaled alcohol seeking and consumption in a way that differs somewhat from sucrose. The authors responded appropriately to prior reviewers. Although there are some definitions and words usage that is not supported, the overall findings are novel. Some additional analyses may serve to strengthen their claims.

The use of the word preference is misleading. I kept waiting for a preference assessment in the home-cage drinking set up where high and low drinkers were split that was never performed. What was done, was examining total 24 h consumption across training and taking a split previously used in the literature. This is not a preference, but a different consumption history, with an arbitrary split. This is a phenotype of slighter higher drinking. Even high drinking in this study is misleading, as rats are barely drinking over a 24 h period. No BECs are reported, and without that, whether the low overall consumption measured would be pharmacologically relevant at any point in a daily cycle is not clear. I appreciate the goal of looking at individual differences, but without more evidence, the claim of high drinkers or higher preference is not supported. Different consumption histories, and total amount of alcohol consumed are different. What is a more likely contributor, is differences in palatability and the ability for experience to enhance any palatability change, and given the use of Long Evans, a reduction in aversion to taste properties of alcohol seems likely. The authors do mention palatability, but there is quite an extensive and literature of OFC and palatability. Whether OFC is directly involved in determining palatability, or represents aspects of palatability and its use may be of interest for alcohol self-administration.

EDITOR’S NOTE: Please revise the text to address this issue of preference and palatability. Please include any preference data, e.g. from home cage testing, if you have it. This is a major issue to focus on for the revised manuscript.

We have included the following statement in the discussion manuscript:

"We note a number of conceptual limitations in this study. We were interested in how OFC neuronal activity differed based on differences in motivation to consume ethanol. We use the term individual preference as a measure of willingness to intake alcohol, measured behaviorally, but we note that preference is frequently characterized by measurements such as comparing ethanol vs. water intake. Because we did not perform simultaneous 2-bottle ethanol vs. water measurements, we cannot conclusively say that the differences in alcohol consumption are preference, per se, but we note that individual variability in alcohol intake, including variability that arises over time during intermittent homecage access, is typically concurrent with alcohol preference as measured in 2-bottle tests. We also note that individual differences in OFC activity (or differences in HD vs. LD rats) may have been driven by differences in overall ethanol intake history. This fascinating question of innate vs. exposure-driven differences in ethanol motivation is something that we are currently exploring. We also acknowledge that levels of drinking performed by high drinking rats here are lower than that observed using other techniques such as high-drinking rat strains, or chronic intermittent exposure to ethanol vapor. Although high drinking rats frequently consumed high levels of ethanol (in some cases over 10 g/kg/24 hours), and were on average, in line with previous reports of high drinking in outbred rats (Simms et al., 2008; George et al., 2012; Carnicella et al., 2014; Momeni and Roman, 2014; Spoelder et al., 2015), we cannot say for sure that drinking in this study was driven by pharmacological elements of ethanol as opposed to taste or palatability aspects, particularly given that we did not measure blood ethanol levels. Given the role of the OFC in processing taste and taste preferences (Rolls 2015), this issue is something that should be addressed in future studies.”

Missing is some reporting on the dynamics observed within a neuron. This is a dynamic task. The activity of neurons is split up by epochs and the average reported, whether it is increasing or decreasing from baseline, and how many neurons showed similar effects. This is then used to build a picture of what “OFC” activity looks like. This is fairly standard for the field, but almost a bit misleading and the take away picture is not clear. Figure 2D was per animal, but I don’t think Figure 3 was. Grouping them this way can be misleading as to what the overall output of OFC may look like. Careful phrasing can reduce this, just saw more down modulation, or more up modulation etc.

EDITOR’S NOTE: I agree that it is hard to judge dynamics as well as variability over neurons, given how you have shown the data. Having one raster is much appreciated, but how does the ensemble average capture the diversity of responses over neurons and the epochs shown in Fig 2D? As you recorded from multiple neurons simultaneously, could you include a new figure showing activity across one or two sets of simultaneously recorded neurons to depict cross neuron variability and temporal dynamics? (This could be made quickly using a program such as NEx.)

There appear to be two important issues (at least) here. The first, raised by the reviewer, is whether or not significantly-modulated neurons exhibited the same pattern of activity, thus generating the population-averaged plots that we have shown. The second, raised by the editor, is whether there were time-locked correlations in neuronal responses in simultaneously-recorded neurons. These points intersect, as correlated activity would indicate a relatively homogeneous coherent signal arising from OFC. Rather than dive into the relatively complex analysis underscoring dynamics of temporal coordination (particularly given the relatively unconstrained nature of the task, which led to significant trial-to-trial variability), we took a simpler approach to address the reviewer’s point. We classified each neuron as significantly excited, inhibited, or neither during each of our three measured epochs. Then, rather than combining the responses, we characterized the proportions of different response profiles (e.g., inhibited in the Cue epoch, excited in the Seeking epoch, inhibited in the reward epoch, etc.). Based on this analysis, shown in Tables 1 (for blocked) and 2 (for interleaved), we found that OFC neuronal responses are heterogeneous, but not completely - i.e., there were specific patterns of activity that were best represented across the recorded population. Interestingly, in blocked conditions, the specific patterns that were best represented differed across outcome time, with 20% EtOH conditions showing the most different categories of response profile. In the interleaved conditions, there was a strong bias towards patterns focused on inhibition during sucrose consumption, unsurprisingly, and a large number of representations of many different categories present in small (1-2) numbers of neurons. These results are included in the revised manuscript, and it is noted that, rather than OFC broadcasting a unified signal, there is substantial heterogeneity in response properties, possibly due to differential connectivity or other aspects of ensemble differentiation.

We note that the question of simultaneous cross-neuron dynamics is of significant interest and is something that needs to be pulled out of this, or related, data sets. However, we decided that the amount of explanation and investigation that would need to be included in the manuscript to address this question would detract from the main goal of associating OFC activity with individual preference for EtOH. Given our interest in neural coding, we plan to address this in follow-up studies, in which the numbers of simultaneously-recorded neurons are greater and the task parameters are more constrained to permit more rigorous temporal analysis.

Outcome signaling or reward signaling is thought of as the ground truth for OFC activity. But here, most neurons that shown modulation are down modulated. Do the authors think of cue or seeking as outcome signaling?

EDITOR’S NOTE: Please revise the text to address this issue.

This is an interesting question. Most studies reporting outcome encoding appear to show differential outcome representation during the cue presentation or during the action related to acquiring the outcome (for example, see references in Sharpe and Schoenbaum 2016, Stalnaker et al., 2015) and have ascribed outcome encoding in OFC as a predictive function. We have seen this suppression during consumption before (Moorman and Aston-Jones 2014) and it has been shown in other studies (van Duuren et al. 2009, Jo and Jung 2016, Lopatina et al. 2017, and probably more). However, to our knowledge there haven’t been too many studies specifically investigating differential activity during consumption, particularly in the rodent. One reason for this is that it is hard to get animals to consume a less-palatable outcome, so differential encoding during consumption is challenging. This, in our opinion, is one of the strengths of our manuscript - showing that consumption-related activity is different depending on the (individually-defined) palatability of the outcome. In some regard, our results show the inverse of what is typically considered value-encoding - stronger inhibition for more preferred outcomes. Exactly what drives this inhibition, and how it relates to differential outcome encoding in a non-alcohol context, is worth separate study in and of itself. We are actually addressing this question in our ongoing work, and we appreciate the suggestion to look into this further. We have also included a mention of this in the discussion.

One thing that did stand out, was the greater down modulation during licking behaviors, at least in the sucrose and the 10% ethanol, less so for 20%. Given that activity analysis was limited to licking periods, and rats drinking 20% licked less and for shorter periods, is OFC activity steady across the period?

EDITOR’S NOTE: Please revise the text to address this issue. You might want to use a sliding window measure of firing rate over the period of reward to address this issue.

The issue raised here is whether or not shorter duration licking for ethanol was too short to allow us to observe a hypothesized natural development of inhibition as seen in sucrose. Thus, the counterargument to our point that OFC activity is differentially modulated based on sucrose vs. ethanol outcome is that, after the initiation of licking, there is a temporally consistent process of inhibition that occurs for all licking behavior that is truncated by shorter ethanol licking times.

To address this issue, we performed a sliding window analysis using 100 ms bins to determine when OFC firing was significantly inhibited during consumption relative to baseline. We performed this analysis on sucrose and 20% ethanol sessions, as these were the extremes with respect to reward consumption duration and because responses were strongly downmodulated and responses in ethanol were not. We calculated, on a neuron-by-neuron basis, the timestamps of the first of two consecutive 100 ms bins following the initiation of consumption that reached statistically significant inhibition compared to baseline. We then compared the relationship of this onset of inhibition to lick durations in both sucrose and ethanol. In sucrose sessions, the median latency for inhibition to develop post-lick was 0.5 seconds. This was not different than that seen in the small number of neurons in which inhibition was observed in 20% ethanol trials (median = 0.7 seconds; p = 0.29, Wilcoxon rank sum). In contrast, the latency of inhibition onset was significantly different, and much shorter than the duration of licking measured in these analyses (sucrose duration median: 6.38 seconds vs. 20% ethanol median 5.47 seconds, all comparisons of inhibition latency vs. consumption duration p<0.0001). So although there was a significant difference between sucrose and 20% ethanol consumption duration, this difference did not overlap with the onset of potential inhibition, as seen in sucrose trials and in the small number of significantly inhibited neurons in ethanol trials. Beyond statistical significance, the overall differences in magnitude (<1 seconds for onset of inhibition vs. >3 seconds for duration of consumption) strongly indicate that there was ample 20% ethanol consumption to permit possible inhibition of OFC activity, if it were to occur. Instead, we conclude that inhibition of OFC activity during sucrose consumption was not present in the majority of neurons recorded during 20% ethanol consumption, independent of duration of licking. These analyses are included in the revised manuscript.

Also please see our response to Reviewer 1, point 2, and the incorporation of additional correlation analyses to address this issue. As noted, we observed no clear relationship between lick duration and OFC activity. In contrast, we saw significant differences during consumption between (relative) high- vs. low-drinking animals.

Taking into account the 5 additional analyses performed to address whether or not differential inhibition was in fact reflective of differential licking behavior, we continue to assert that differences in OFC activity are more related to innate differences in motivation for/preference for/palatability of ethanol, as opposed to licking behavior, lack of movement during consumption, or other alternate explanations (that we have investigated).

One of the section titles seems to be misleading; was there an analysis that showed home cage ethanol preference predicted ethanol seeking? That would be a correlation? Group differences are shown, but we don’t know about prediction.

EDITOR’S NOTE: Please revise the text to address this issue.

This has been changed to “...was associated with...”

In this frame work, simple correlations between total change in modulation predict longer bout sizes or more licks?

EDITOR’S NOTE: Please revise the text to address this issue. I would like to suggest that you use Spearman rank correlation to examine relationships between firing rate and bout duration + lick count per trial.

Please see our response to Reviewer 1, point 2, and the incorporation of additional analyses to address this issue. As noted, we observed no clear relationship between lick duration and OFC activity. We used Spearman rank correlation in these new analyses.

Some care should be taken with wording. For example, OFC neurons treating ethanol and sucrose as less palatable in the interleaved trials in LD rats (last sentence of results and in discussion as well). I don’t believe there is supporting data for that claim; as interleaved trials could exert a lot of other effects other than an anticipatory contrast effect on consumption. Even that, isn’t necessarily tied to palatability.

EDITOR’S NOTE: Please revise the text to address this issue.

This has been changed in the results and discussion to emphasize that this is one, but not the only, possibility.

In the discussion, newly added wording about cue activity differing not explained by behavior because there was similar nose-poke demands, is not correct. Just because the rules were the same across conditions, does not mean the behavior emitted and the time course with which it is emitted are.

EDITOR’S NOTE: Please revise the text to address this issue.

This statement has been removed from the discussion.
